# Comprehensive analysis of the m6A-related molecular patterns and diagnostic biomarkers in osteoporosis

**DOI:** 10.3389/fendo.2022.957742

**Published:** 2022-08-10

**Authors:** Qiong Bai, Min Shi, Xinli Sun, Qiu Lou, Hangya Peng, Zhuan Qu, Jiashuang Fan, Lifen Dai

**Affiliations:** ^1^ Laboratory of Genetic Breeding and Molecular Biology, Southwest Forestry University, Kunming, China; ^2^ National Wetland Ecosystem Fixed Research Station of Yunnan Dianchi, Southwest Forestry University, Kunming, China; ^3^ Department of Internal Medicine, The Affiliated Hospital of Yunnan University, Kunming, China; ^4^ Department of Internal Medicine, Yunnan Fuwai Cardiovascular Hospital, Kunming, China; ^5^ Department of Internal Medicine, The Second Affiliated Hospital of Kunming Medical University, Kunming, China

**Keywords:** N6-methyladenosine, Osteoporosis, molecular patterns, WGCNA, diagnostic markers

## Abstract

**Background:**

N6-methyladenosine (m6A) modification is a critical epigenetic modification in eukaryotes and involves several biological processes and occurrences of diseases. However, the roles and regulatory mechanisms of m6A regulators in osteoporosis (OP) remain unclear. Thus, the purpose of this study is to explore the roles and mechanisms of m6A regulators in OP.

**Methods:**

The mRNA and microRNA (miRNA) expression profiles were respectively obtained from GSE56815, GSE7158, and GSE93883 datasets in Gene Expression Omnibus (GEO). The differential expression of 21 m6A regulators between high-bone mineral density (BMD) and low-BMD women was identified. Then, a consensus clustering of low-BMD women was performed based on differentially expressed (DE)-m6A regulators. The m6A-related differentially expressed genes (DEGs), the differentially expressed miRNAs (DE-miRNAs), and biological functions were investigated. Moreover, a weighted gene co-expression network analysis (WGCNA) was constructed to identify the OP-related hub modules, hub genes, and the functional pathways. Then, an m6A regulator–target–pathway network and the competing endogenous RNA (ceRNA) network in key modules were constructed. A least absolute shrinkage and selection operation (LASSO) Cox regression model and a Support Vector Machine-Recursive Feature Elimination (SVM-RFE) model were constructed to identify the candidate genes for OP prediction. The receiver operator characteristic (ROC) curves were used to validate the performances of predictive models and candidate genes.

**Results:**

A total of 10,520 DEGs, 13 DE-m6A regulators, and 506 DE-miRNAs between high-BMD and low-BMD women were identified. Two m6A-related subclusters with 13 DE-m6A regulators were classified for OP. There were 5,260 m6A-related DEGs identified between two m6A-related subclusters, the PI3K-Akt, MAPK, and immune-related pathways, and bone metabolism was mainly enriched in cluster 2. Cell cycle-related pathways, RNA methylation, and cell death-related pathways were significantly involved in cluster 1. Five modules were identified as key modules based on WGCNA, and an m6A regulator–target gene–pathway network and the ceRNA network were constructed in module brown. Moreover, three m6A regulators (FTO, YTHDF2, and CBLL1) were selected as the candidate genes for OP.

**Conclusion:**

M6A regulators play an important role in the occurrences and diagnosis of OP.

## Introduction

Osteoporosis (OP), one of the most common bone system diseases, affects older adults by increasing the risk of bone fractures, leading to many complications (1). OP is defined as a skeletal disorder characterized by reduced bone strength (bone density and bone quality), which increases the risk of fracture (2). With the World Trade Organization (WTO) criteria, people with bone mineral density (BMD) of less than −2.5 standard deviations (SDs) are defined as having OP (3, 4). In recent decades, improving life and medical conditions are the leading cause of the average life expectancy. According to the WHO, the elderly population worldwide will reach 12 billion in 2025, and approximately 70% will be found in developing counties (5). A study has shown that the elderly population of over 65 years are 91.5 million in 2020 and will be expected to reach 183.6 million by 2024 in China (6). The Chinese population with OP is 83.9 million in 1997 and is expected to reach 212 million by 2050 (7). OP has become a serious public health problem in China, especially among elderly postmenopausal women (8, 9). There are two current important approaches for the prevention and treatment of OP, fundamental supplement for bone health and pharmacological treatment, both of which are expensive, thus increasing the familial and societal economic burden of OP and OP-related fracture (9, 10). Based on this, there is a need to discover novel biomarkers for early diagnosis and therapy of OP.

In the past decade, the studies have shown the pathogenesis of OP links to processes at the tissue, cellular, and molecular levels (11), which involve osteoblast–osteoclast differentiation (12), bone metabolism (13), and bone immunity (14, 15). The communication and crosstalk between the main bone cell types discover the pathogenesis at the cellular level. Several molecules exert biological function during bone remodeling, such as microRNA-241 (miR-241) and its target ATF4 (16), prostaglandin E2 (IGE2) (17), and semaphorin 3A (18). The research of cellular and molecular levels supplements the concept of bone pathophysiology and possibly provides breakthrough advances in clinical practice.

N6-methyladenosine (m6A) modification is one of the most common epigenetic modifications in eukaryotic mRNAs and involves mammalian development and disease control by regulating RNA processing and metabolism (19, 20). The M6A modification process is catalyzed by highly conserved methyltransferases (writers), demethylases (erasers), and binding proteins (readers) (21, 22). M6A modification widely occurs in mRNAs, long non-coding RNAs (lncRNAs), and miRNAs in many eukaryotes (23, 24). Increasing evidence has suggested that m6A modification acts as a novel epitranscriptomic marker and exerts a dominant role in bone development and metabolism of OP (25, 26). For example, FTO-mediated m6A demethylation in the 3’UTR of PPARG mRNA promotes osteogenic differentiation of mesenchymal stem cells (CSCs) (27). Peng Jun et al. have found that METTL3-mediated m6A methylation of LINC00657 promotes the development of osteogenesis, and LINC00657 functions as a ceRNA to upregulate BMPR1B *via* sponging miR-144-3p (28). Increasing evidence reveals that m6A-related lncRNA, miRNAs, and mRNA exert the domain roles in the development of OP, which may serve as the novel potential targets for diagnosis and therapeutic targets for OP. However, the biological significance of the m6A regulators in OP remains elusive.

In the present study, we aimed to investigate the biological roles and regulatory mechanisms in OP. To achieve this goal, we comprehensively explored the functions of m6A regulators in the molecular pattern classification, regulatory mechanisms, and diagnosis of OP based on the gene expression profiles from Gene Expression Omnibus (GEO). We not only constructed the m6A-related subclusters and an m6A–target–pathway network but also selected three candidate m6A regulators for diagnosis of OP.

## Materials and methods

### Data collection and processing

The microarray data and corresponding clinical information of female patients with low or high BMD were downloaded from GSE7158 and GSE56815 datasets in the GEO database (**Table S1**) (https://www.ncbi.nlm.nih.gov/geo/). In detail, the GSE56815 dataset was generated by the GPL96 platform and contained 40 high (20 pre- and 20 postmenopausal) and 40 low hip BMD (20 pre- and 20 postmenopausal) monocyte samples. The GSE7158 dataset was generated by the GPL570 platform and contained 14 high and 12 low hip BMD monocyte samples. The specific miRNA profiles with 12 OP (6 OP patients with vertebral fractures and 6 OP patients without vertebral fractures) and 6 non-OP patients and their corresponding clinical information were obtained from the GEO database (**Table S1**), generated by the GPL18058 platform. Limma R package was performed to screen the DEGs between high-BMD and low-BMD samples, and the DE-miRNAs. *p*-value < 0.05 was considered to be the cutoff criterion for the identification of DEGs or DE-miRNAs.

### Screening of the DE-m6A regulators

A total of 21 m6A regulators, including eight writers (METTL3, ZC3H13, METTL14, RBM15B, CBLL1, WTAP, RBM15, and KIAA1429), two erasers (FTO and ALKBH5), and 11 readers (YTHDC1, YTHDC2, ELAVL1, YTHDF1, LRPPRC, YTHDF2, FMR1, YTHDF3, HNRNPC, HNRNPA2B1, and IGF2BP1), were selected to explore the DE-m6A regulators between high-BMD and low-BMD samples.

### Consensus clustering analysis

Consensus clustering is an unsupervised clustering method that was applied to class discovery and clustering (29). Here, the ConsensusClusterPlus R package was used to class the low-BMD samples into different subgroups according to the DE-m6A regulators. ConsensusClusterPlus R was performed 1,000 times to guarantee the stability of the classification. The number of clusters k was determined by the consensus clustering cumulative distribution function (CDF). Furthermore, t-distributed Stochastic Neighbor Embedding (t-SNE) is a dimension reduction method to reveal population stratification at different scales (30), and is used to verify the classification performance based on the mRNA expression profile of the above DE-m6A regulators.

### Identification of the m6A-related DEGs between subgroups and functional enrichment analysis

Limma R package was performed to filter the m6A-related DEGs between two subgroups based on the cutoff values of *p*-value < 0.05. GSEA is a bioinformatic approach for investigating statistically significant and concordant differences between two groups based on a prior defined set of genes (31). In the present study, GSEA was used for GO and KEGG pathway enrichment. The significantly enriched pathways were identified according to the threshold value of *p*-value < 0.05 and |normalized enrichment score (NES)| > 1. Based on the GSEA results, we identified the important signaling pathways in OP, including cell cycle, apoptosis, methylation, metabolism, immunity, and osteoclast differentiation. Then, the GSVA algorithm was performed using the Limma R package to probe into the distinct signaling pathways between two subgroups. Differential signaling pathways were identified with the criteria of *p*-value < 0.05 and |*t*| > 1.

### WGCNA

WGCNA is a bioinformatics method for describing the correlation patterns among genes across microarray samples and can be used for finding candidate biomarkers or therapeutic targets in various biological contexts (32). WGCNA R package was used to identify the hub genes and the low-BMD-related modules among low-BMD samples in the GSE56815 dataset. Firstly, we evaluated the availability of the genes across low-BMD samples and subsequently constructed an adjacency matrix to describe the correlation strength between the nodes. Here, we chose a soft-threshold *β* = 8 (scale-free *R* ^2^ = 0.85). Secondly, the adjacency matrix was transformed into a topological overlap matrix (TOM) to quantitatively describe the similarity in nodes. Thirdly, we performed hierarchical clustering to identify the modules with a minimum size of 30 genes.

### Identification of the low-BMD-related modules and significant targets

After calculating the module eigengene (ME), we hierarchically clustered the modules and then merged similar modules. We explored the significant modules relevant to low-BMD based on the gene significance (GS) and module membership (MM), which respectively represent the biological significance and correlation between the gene expression profile and the ME. We identify the significant low-BMD-related modules according to the correlation coefficient > 0.5 and *p*-value < 0.05. Then, we downloaded the m6A target gene sets from the M6A2Target database (http://m6a2target.canceromics.org/), which is a comprehensive database for targets of m6A writers, erasers, and readers (33). The correlation between m6A target genes and the DE-m6A regulators in low-BMD samples was calculated, and the significant m6A regulators and their target genes were identified according to the correlation coefficient > 0.3 and *p*-value < 0.05. Then, the significant target genes were enriched in each module, and the enrichment results were tested using a hypergeometric test (34). We filtered the wear-relevant target genes by setting the cutoff values of MM < 0.3 and GS < 0.3.

### GO annotation and KEGG enrichment analysis

The genes in the significantly related module were selected to conduct GO and KEGG enrichment analysis using the clusterProfile R package. The significant pathways were determined by *p*-value < 0.05.

### Construction of an m6A regulator–target–module–pathway network and the ceRNA network

Based on the above results, an m6A regulator–target–module–pathway network was constructed using Cytoscape software version 3.7.2. According to the DEGs and DE-miRNAs between OP and non-OP, the miRNA–mRNA and miRNA–lncRNAs interactions were predicted using miRcode (http://mircode.org/) (35). The ceRNA network was visualized using the Cytoscape software version 3.7.2. Then, we identified the key ceRNA network that contained the m6A regulators and targets from the previous m6A regulator–target–module–pathway network.

### LASSO regression and SVM-RFE analyses

Least absolute shrinkage and selection operation (LASSO) and Support Vector Machine-Recursive Feature Elimination (SVM-RFE) algorithms were conducted to find the significant prognostic biomarkers in OP. LASSO is a regression analysis algorithm used to filter the variables to prevent overfitting (36). Based on the expression of DE-m6A regulators, the prognostic genes were identified by LASSO regression analysis using the glmnet R package with the penalty parameter (*λ*) tuning conducted by 10-fold cross-validation. Moreover, SVM-RFE is a supervised machine-learning technique to identify the most relevant features by deleting the feature vector generated by SVM (37, 38). Here, the SVM-RFE algorithm was performed using the e1071 R package and used to screen the best variables. Finally, the candidate prognostic genes for OP were obtained by overlapping the candidate genes from two algorithms. The ROC curves were drawn using the pROC R package to verify the predictive performance of candidate genes in the GSE56815 and GSE7158 datasets. The area under the ROC curve (AUC) values were used to estimate the accuracy and efficiency of the candidate genes.

### Statistical analysis

All statistical analyses in this study were performed using R (version 4.0.2). Wilcoxon rank-sum test was conducted to compare differences between groups. A two-tailed *p*-value < 0.05 was considered statistically significant.

## Results

### Identification of the 13 DE-m6A regulators

**Figure 1:**
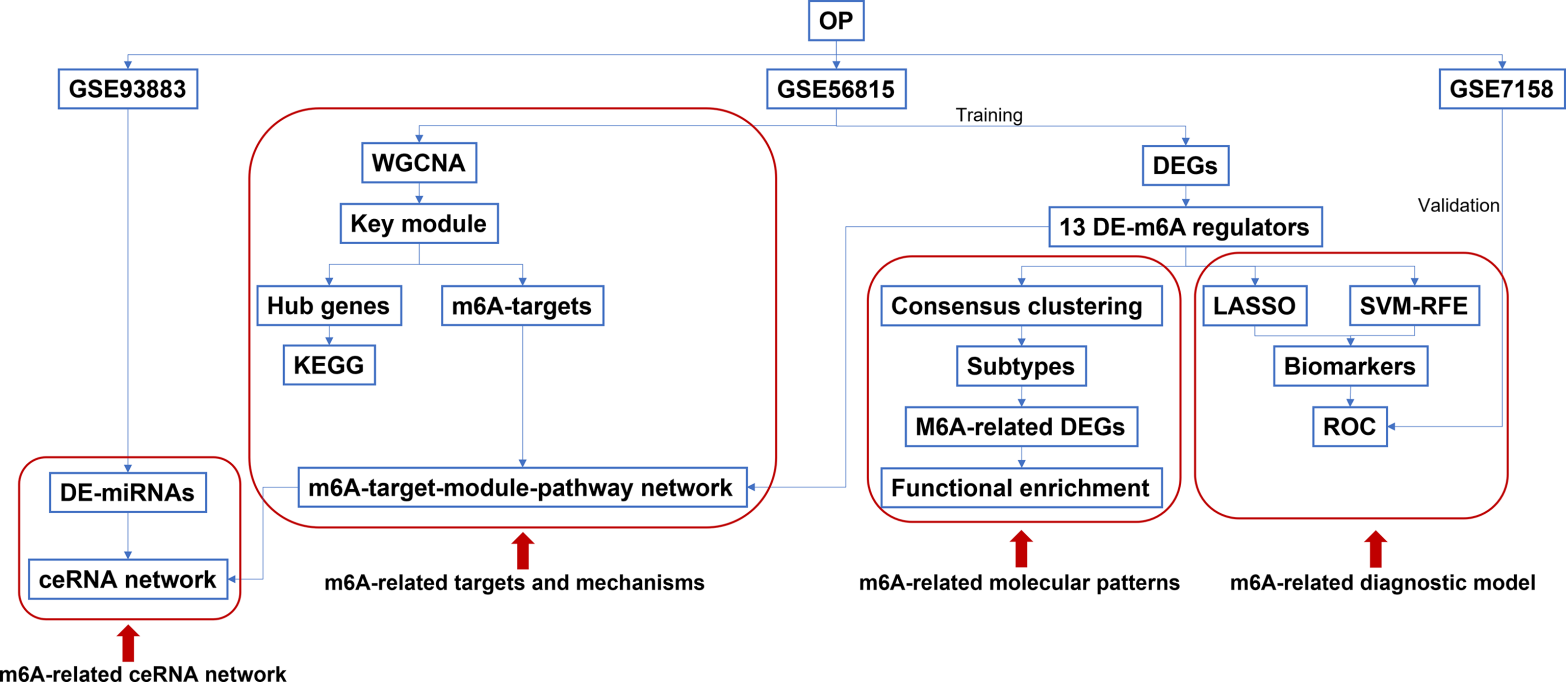
The workflow of the study design.

**Figure 2:**
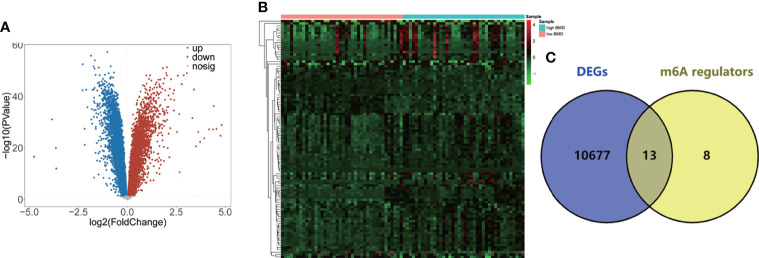
Identification of the 13 DE-m6A regulators. **(A)** Volcano plot showing the DEGs between high-BMD women (*n* = 40) and low-BMD women (*n* = 40) in the GSE56815 dataset. **(B)** Heatmap indicating the top 100 DEGs between high-BMD women (*n* = 40) and low-BMD women (*n* = 40) in the GSE56815 dataset. **(C)** Venn plot showing the DE-m6A regulators between high-BMD women (*n* = 40) and low-BMD women (*n* = 40) in the GSE56815 dataset.

Details of this study are illustrated in **Figure 1**. Based on the GSE56815 dataset, the 10,520 DEGs (4,088 upregulated and 6,432 downregulated DEGs) between high-BMD and low-BMD women were identified using the Limma R package with *p*-value < 0.05 (**Figure 2A**, **Table S2**). The top 100 significant DEGs (74 upregulated and 26 downregulated DEGs) are shown in **Figure 2B**. Moreover, the 13 DE-m6A regulators (METTL3, HNRNPC, FTO, LRPPRC, YTHDC1, YTHDF1, ZC3H13, RBM15, YTHDF3, FMR1, RBM15B, YTHDF2, and CBLL1) between high-BMD and low-BMD women were obtained by overlapping 10,520 DEGs and 21 m6A regulators (**Figure 2C**). These findings suggested that m6A methylation might be involved in the dysregulated BMD in OP.

### Classification of two m6A-related molecular subclusters for OP based on 13 DE-m6A regulators

**Figure 3:**
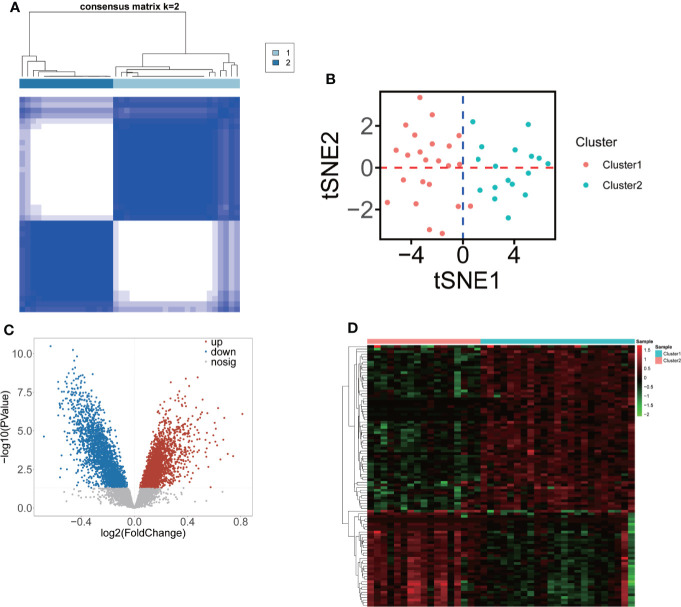
Classification of two m6A-related molecular subclusters for OP based on 13 DE-m6A regulators. **(A)** Heatmap showing the consensus clustering of m6A-related subclusters (*k* = 2) of OP based on 13 DE-m6A regulators. **(B)** The t-SNE plot showing the two clustered samples in the OP. Red represents cluster 1, and blue represents cluster 2. **(C)** Volcano plot showing the DEGs between two m6A-related subclusters. **(D)** Heatmap indicating the top 100 DEGs between two m6A-related subclusters.

Consensus clustering was performed to identify the m6A-related molecular subclusters for OP. As shown in **Figures S1A–I**, the relative change of the cumulative distribution function (CDF) and the area under the CDF curve of the consensus cluster from k = 2 to 6. k = 2 was proven to be the most suitable clustering to divide the 40 low-BMD patients into clusters (cluster 1 = 23, and cluster 2 = 17, **Figure 3A**, **Table S3**). The t-SNE plot indicated that 13 significant m6A regulators could completely distribute the two m6A-related subclusters (**Figure 3B**). A total of 5,260 m6A-related DEGs (2,969 upregulated and 2,291 downregulated m6A-related DEGs) were identified between the two m6A-related subclusters (**Figure 3C**, **Table S4**). The top 100 m6A-related DEGs (37 upregulated and 63 downregulated m6A-related DEGs) are shown in **Figure 3D**.

### Functional analyses of the m6A-related DEGs

**Figure 4:**
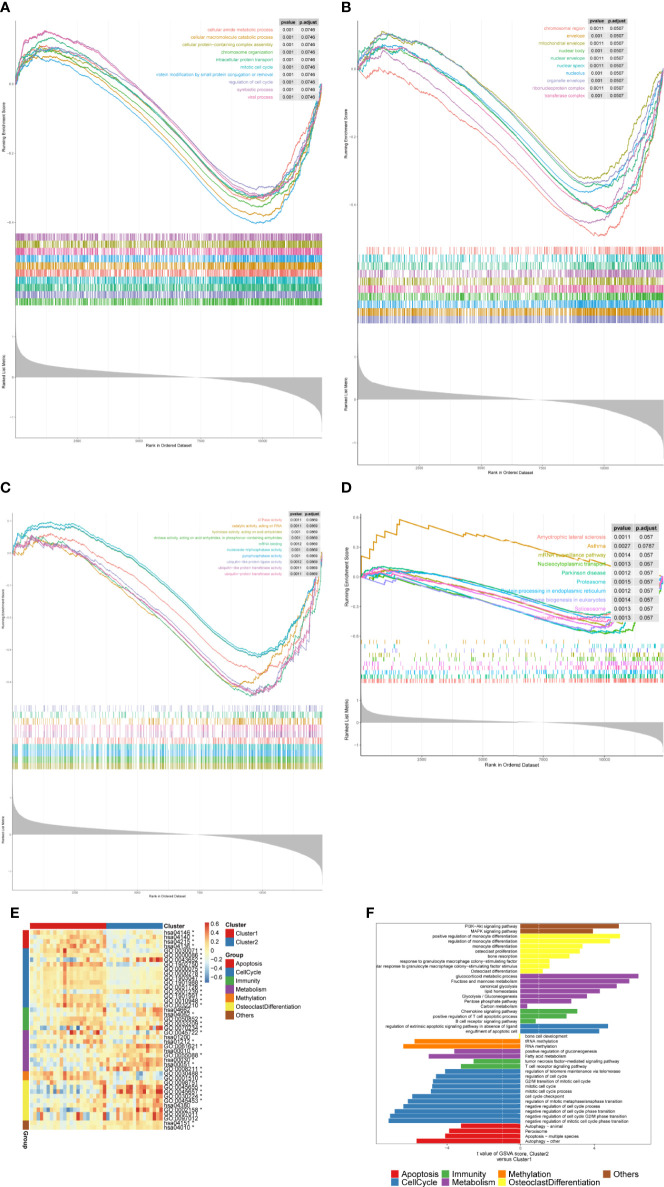
Functional analyses of the m6A-related DEGs. **(A–D)** The GSEA curves showing the GO (BP, CC, and MF) and KEGG pathways between two m6A-related subclusters. **(E, F)** Heatmap and bar charts showing the specific signaling pathways in two m6A-related subclusters.

We further performed the GSEA to investigate the potential mechanism of the m6A-related DEGs in OP. The GO and KEGG pathway enrichment analysis indicated that a total of 2,046 GO terms and 100 KEGG pathways were obtained (**Table S5**). The GO functional enrichment analysis revealed that m6A-related DEGs were mainly involved in chromosome organization, regulation of cell cycle, intracellular protein transport, cellular macromolecule catabolic process, organelle envelope, envelope, hydrolase activity, acting on acid anhydrides, triphosphatase activity, ATPase activity, etc. (**Figures 4A–C**, **Table 1**). Moreover, the KEGG results showed that these genes were significantly associated with protein processing in the endoplasmic reticulum, ubiquitin-mediated proteolysis, spliceosome, nucleocytoplasmic transport, mRNA surveillance pathway, ribosome biogenesis eukaryotes, and proteasome (**Figure 4D**, **Table 2**). Interestingly, we found several OP-related pathways based on the GSEA results, such as apoptosis, cell cycle, immunity, lipid and glucose metabolism, methylation, osteoclast differentiation, and other pathways (**Table S6**). Thus, GSVA was performed to explore the different signaling pathways between two subclusters. The enrichment scores of pathways in each cluster were calculated (**Table S7**), and a total of 22 pathways were upregulated in cluster 2 compared with cluster 1, whereas 22 pathways were downregulated in cluster 2 than cluster 1 (**Figures 4E** **, F**, **Table S7**). The 22 upregulated pathways in cluster 2 included PI3K-Akt and MAPK signaling pathways, positive regulation or regulation of monocyte differentiation, monocyte differentiation, osteoclast proliferation, bone resorption, response to granulocyte-macrophage colony-stimulating factor, cellular response to granulocyte-macrophage colony-stimulating factor stimulus, osteoclast differentiation, glucocorticoid metabolic process, fructose and mannose metabolism, canonical glycolysis, lipid homeostasis, glycolysis/gluconeogenesis, pentose phosphate pathway, carbon metabolism, chemokine signaling pathways, positive regulation of T-cell apoptotic process, B-cell receptor signaling pathway, regulation of extrinsic apoptotic signaling pathway in absence of ligand, and engulfment of apoptosis cell. The 22 downregulated pathways in cluster 2 included bone cell development, RNA or tRNA methylation, positive regulation of gluconeogenesis, fatty acid metabolism, tumor necrosis factor-mediated signaling pathway, T-cell receptor signaling pathway, regulation of telomere maintenance *via* telomerase, regulation of cell cycle, G2/M transition of the mitotic cell cycle, mitotic cell cycle, mitotic cell cycle process, cell cycle checkpoint, regulation of mitotic metaphase/anaphase transition, negative regulation of cell cycle phase transition, negative regulation of cell cycle G2/M phase transition, negative regulation of mitotic cell cycle phase transition, autophagy, apoptosis, and peroxisome.

**Table 1:** Top 10 biological process (BP), cellular component (CC), and molecular function (MF) terms included in GO terms in OP (|NES| > 1, *p*-wfi 2value < 0.05, *q*-value < 0.25).

ID	Description	Set Size	Enrichment Score	NES	*p*-value	*q*-value
**BP**						
GO:0051276	Chromosome organization	956	−0.356044593	−1.5642	0.001013	0.065091
GO:0051726	Regulation of cell cycle	947	−0.306894939	−1.34803	0.001015	0.065091
GO:0006886	Intracellular protein transport	899	−0.329126874	−1.44317	0.001016	0.065091
GO:0044265	Cellular macromolecule catabolic process	870	−0.382664912	−1.67501	0.001017	0.065091
GO:0044403	Symbiotic process	848	−0.326383911	−1.42922	0.001018	0.065091
GO:0000278	Mitotic cell cycle	831	−0.327102205	−1.43219	0.001017	0.065091
GO:0043603	Cellular amide metabolic process	816	−0.335312476	−1.46838	0.001017	0.065091
GO:0034622	Cellular protein-containing complex assembly	814	−0.339337732	−1.48557	0.001018	0.065091
GO:0016032	Viral process	807	−0.331699238	−1.45128	0.001018	0.065091
GO:0070647	Protein modification by small protein conjugation or removal	805	−0.404121067	−1.76861	0.001017	0.065091
**CC**						
GO:0031967	Organelle envelope	864	−0.338298657	−1.49285	0.001022	0.038992
GO:0031975	Envelope	864	−0.338298657	−1.49285	0.001022	0.038992
GO:0005730	Nucleolus	700	−0.364976689	−1.60546	0.001028	0.038992
GO:0016604	Nuclear body	633	−0.423652483	−1.85605	0.001043	0.038992
GO:1990234	Transferase complex	579	−0.41297279	−1.8024	0.001047	0.038992
GO:0005740	Mitochondrial envelope	515	−0.326814516	−1.42025	0.001057	0.038992
GO:1990904	Ribonucleoprotein complex	504	−0.455696573	−1.97396	0.001067	0.038992
GO:0005635	Nuclear envelope	372	−0.3500178	−1.49069	0.0011	0.038992
GO:0016607	Nuclear speck	329	−0.428431923	−1.80671	0.001122	0.038992
GO:0098687	Chromosomal region	280	−0.494054321	−2.05569	0.001139	0.038992
**MF**						
GO:0016817	Hydrolase activity, acting on acid anhydrides	629	−0.324416155	−1.41125	0.001037	0.074647
GO:0016818	Hydrolase activity, acting on acid anhydrides, in phosphorus-containing anhydrides	629	−0.324416155	−1.41125	0.001037	0.074647
GO:0016462	Pyrophosphatase activity	626	−0.323272875	−1.40599	0.001038	0.074647
GO:0017111	Nucleoside-triphosphatase activity	588	−0.316533271	−1.37166	0.001047	0.074647
GO:0016887	ATPase activity	318	−0.376932559	−1.5878	0.001124	0.074647
GO:0019787	Ubiquitin-like protein transferase activity	290	−0.442362866	−1.84804	0.001143	0.074647
GO:0004842	Ubiquitin-protein transferase activity	272	−0.437820575	−1.82051	0.001144	0.074647
GO:0140098	Catalytic activity, acting on RNA	266	−0.415825581	−1.72632	0.001145	0.074647
GO:0003729	MRNA binding	222	−0.444648281	−1.81033	0.001183	0.074647
GO:0061659	Ubiquitin-like protein ligase activity	183	−0.43076598	−1.72056	0.001222	0.074647

**Table 2:** Top 10 KEGG pathways in OP (|NES| > 1, *p*-value < 0.05, *q*-value < 0.25).

ID	Description	Set Size	Enrichment Score	NES	*p*-value	*q*-value
hsa05014	Amyotrophic lateral sclerosis	278	−0.38971	−1.6096	0.001133	0.042387
hsa05012	Parkinson disease	200	−0.39188	−1.57062	0.001185	0.042387
hsa04141	Protein processing in endoplasmic reticulum	145	−0.42183	−1.64561	0.001205	0.042387
hsa04120	Ubiquitin-mediated proteolysis	121	−0.45869	−1.74585	0.001252	0.042387
hsa03040	Spliceosome	102	−0.51279	−1.9074	0.001302	0.042387
hsa03013	Nucleocytoplasmic transport	90	−0.57872	−2.13247	0.001319	0.042387
hsa03015	mRNA surveillance pathway	72	−0.50116	−1.78817	0.001377	0.042387
hsa03008	Ribosome biogenesis in eukaryotes	53	−0.54982	−1.84501	0.001437	0.042387
hsa03050	Proteasome	42	−0.5757	−1.84178	0.001504	0.042387
hsa05310	Asthma	25	0.579696	1.844009	0.002681	0.05852

### Identification of the OP-related modules by WGCNA

**Figure 5:**
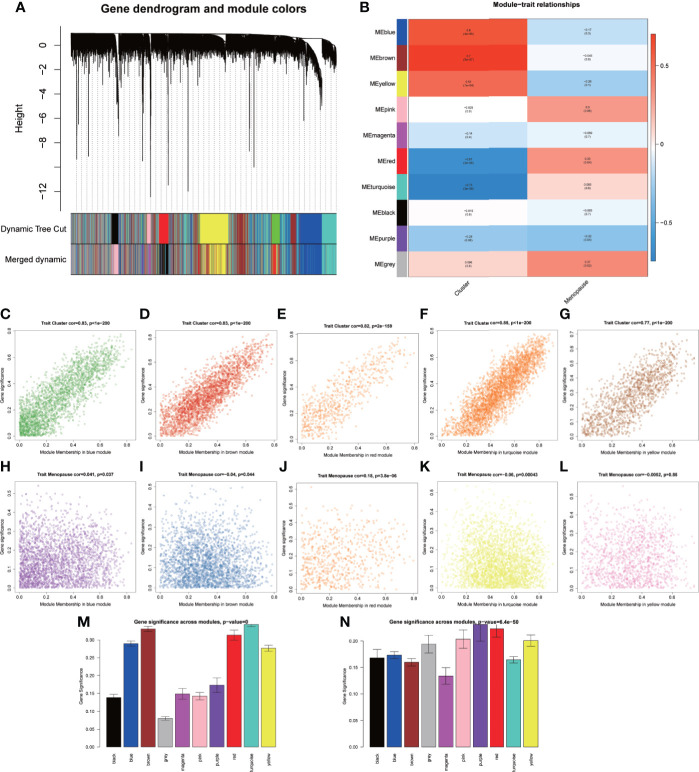
Identification of the OP-related modules by WGCNA. **(A)** Clustering dendrogram of genes based on the measurement of dissimilarity (1-TOM) together with the assigned module colors. **(B)** Heatmap showing the correlation between the module eigengenes and clinical traits of OP. **(C–G)** Scatterplots showing the correlation between the MM and GS in each module (MEblue, MEbrown, MEyellow, MEred, and MEturquoise). **(H–L)** Scatter plots showing the correlation between the MM and clinical trait (menopause) in each module (MEblue, MEbrown, MEyellow, MEred, and MEturquoise). **(M)** Bar charts showing the correlation between GS and cluster trait in each module (MEblue, MEbrown, MEyellow, MEred, and MEturquoise). **(N)** Bar charts showing the correlation between GS and clinical trait (menopause) in each module (MEblue, MEbrown, MEyellow, MEred, and MEturquoise).

We also want to identify the meaningful modules that are most associated with OP and low-BMD. Thus, we performed WGCNA with the expression profile of 12,403 genes from 40 samples in the GSE56815 cohort as the input to search for OP and low-BMD-specific genes (**Figure S2A**, **Table S8**). A power *β* = 8 was selected as the soft threshold for scale-free network construction (**Figure S2B**). Ten modules were identified by clustering dendrogram (**Figure 5A**, **Figure S2C, D**). Considering the close correlation between modules and menopause, five modules (MEblue, *r* = 0.6, *p* = 4e-05; MEbrown, *r* = 0.7, *p* = 5e-07; MEyellow, *r* = 0.51, *p* = 7e-04; MEred, *r* = −0.67, *p* = 3e-06; MEturquoise, *r* = −0.75, *p* = 3e-08) were identified in the hub modules for OP and low BMD (**Figure 5B**, **Table S8**). Therefore, MEblue, MEbrown, MEyellow, MEred, and MEturquoise modules were selected for subsequent analyses. We then investigated the correlation between the module membership and OP and low-BMD-related gene significance, or that between menopause and OP and low-BMD-related gene significance, which suggested that the expression levels of OP and low-BMD-related genes within the five modules influenced the OP (**Figures 5C** **–G, M**). However, the expression levels of OP and low-BMD-related genes tended to not be directly influenced by menopause (**Figures 5H–L, N**).

### Enrichment analysis of the MEbrown module

**Figure 6:**
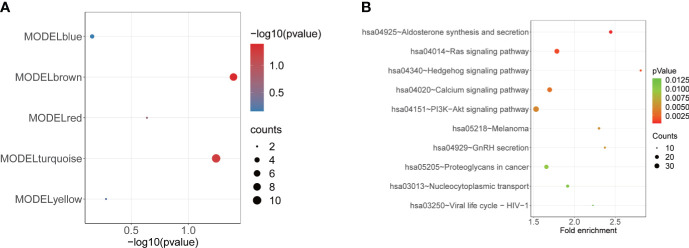
Enrichment analysis of the MEbrown module. **(A)** Bubble plot showing the enrichment of m6A targets in each module (MEblue, MEbrown, MEyellow, MEred, and MEturquoise). **(B)** Bubble plot indicating the KEGG pathways of MEbrown.

To estimate the effect of m6A regulators on the low-BMD of OP, we obtained the 198 m6A targets for the previous seven DE-m6A regulators (**Table S9**) from the m6A2Target database (http://m6a2target.canceromics.org/) to subsequent analyses. We identified the strong correlation between five m6A regulators (METTL3, YTHDF2, YTHDC1, FTO, and HNRNPC) and 39 m6A targets with the threshold of *r* > 0.3 and *p*-value < 0.05 (**Table S9**). Additionally, we also filtered the m6A targets with a weak correlation connected to modules with a threshold of MM < 0.3 and GS < 0.3. Then, a hypergeometric test was performed to identify the enrichment of m6A targets in each module, which suggested that m6A targets were significantly enriched in module brown (**Figure 6A**, **Table 3**). Furthermore, GO and KEGG pathway enrichment analyses were performed to identify the potential biological functions of module brown-related genes, resulting in a total of 36 pathways being dysregulated in module brown with *p* < 0.05 (**Table S10**). The most significant pathways are shown in **Figure 6B** and **Table 4**, and the genes in module brown were enriched in aldosterone synthesis and secretion, Ras signaling pathway, Hedgehog signaling pathway, calcium signaling pathway, PI3K-Akt signaling pathway, GnRH secretion, proteoglycans in cancer, nucleocytoplasmic transport, viral life cycle–HIV-1, and melanoma.

**Table 3:** Enrichment of 39 targets of 13 m6A regulators in five modules.

Model	*p*-value	Counts
MODELblue	0.6968	3
MODELbrown	0.0401	8
MODELred	0.2302	2
MODELturquoise	0.0570	10
MODELyellow	0.5262	2

**Table 4:** Top 10 KEGG pathways in module brown (*p*-value< 0.05).

ID	Description	Counts	*p*-value	FDR
has04925	Aldosterone synthesis and secretion	17	0.000468	0.14834
has04014	Ras signaling pathway	30	0.001304	0.151088
has04340	Hedgehog signaling pathway	11	0.00143	0.151088
has04020	Calcium signaling pathway	29	0.003372	0.260516
has04151	PI3K-Akt signaling pathway	39	0.004807	0.260516
has05218	Melanoma	12	0.005177	0.260516
has04929	GnRH secretion	11	0.005753	0.260516
has05205	Proteoglycans in cancer	24	0.009709	0.34908
has03013	Nucleocytoplasmic transport	15	0.010756	0.34908
has03250	Viral life cycle–HIV-1	10	0.012892	0.34908

### Construction of an m6A regulator–target gene–pathway network and the ceRNA network

**Figure 7:**
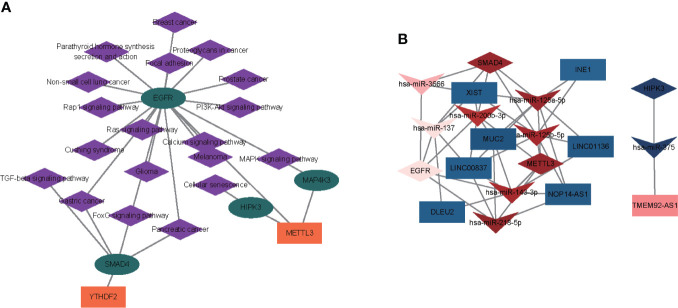
Construction of an m6A regulator–target gene–pathway network and the ceRNA network. **(A)** A m6A regulator–target–pathway network in MEbrown. Orange rectangles present m6A regulators, turquoise ellipses represent m6A targets, and purple diamonds represent pathways. **(B)** Two ceRNA networks of OP. One network contained lncRNA TMEM92-AS1, has-miR-375, and HIPK3. Another ceRNA network included seven lncRNAs (XIST, MUC2, NOP14-AS1, INE1, LINC01136, LINC00837, and DLEU2), seven miRNAs (hsa-miR-125a-5p, hsa-miR-125b-5p, hsa-miR-137, hsa-miR-143-3p, hsa-miR-200b-3p, hsa-miR-218-5p, and hsa-miR-3666), and three mRNAs (SMAD4, METTL3, and EGFR). Rectangles represent lncRNAs, quadrangles represent miRNAs, and prisms represent mRNAs.

Based on enrichment analysis and m6A target investigation in module brown, we constructed a strong correlated m6A regulator–target gene–pathway network based on two m6A regulators (METTL3 and YTHDF2), four m6A targets (SMAD4, HIPK3, MAP4K3, and EGFR), and 19 signaling pathways (prostate cancer, breast cancer, non-small cell lung cancer, MAPK signaling pathway, Cushing syndrome, glioma, melanoma, gastric cancer, pancreatic cancer, FoxO signaling pathway, PI3K-Akt signaling pathway, focal adhesion, parathyroid hormone synthesis, Rap1 signaling pathway, Ras signaling pathway, calcium signaling pathway, proteoglycans in cancer, cellular senescence, and TGF-beta signaling pathway) (**Figure 7A**, **Table S11**). To explore the molecular mechanism of m6A regulators in OP, we further investigated whether the m6A regulators and m6A-related genes were regulated by lncRNA and miRNA. A total of 506 DE-miRNAs between OP and non-OP were identified with a *p*-value < 0.05 (**Table S12**). Based on 10,520 DEGs and 506 DE-miRNAs, 656 miRNA–lncRNA pairs and 99 miRNA–mRNA pairs were identified, which included 65 miRNAs, 71 lncRNAs, and 4 mRNAs (**Table S13**). Then, we selected the key ceRNA network by inserting four mRNAs and six genes (METTL3, YTHDF2, SMAD4, HIPK3, MAP4K3, and EGFR) from the above network. The two key ceRNA networks were obtained (**Figure 7B**, **Table S13**). A ceRNA network contained lncRNA TMEM92-AS1, has-miR-375, and HIPK3. Another ceRNA network included seven lncRNAs (XIST, MUC2, NOP14-AS1, INE1, LINC01136, LINC00837, and DLEU2), seven miRNAs (hsa-miR-125a-5p, hsa-miR-125b-5p, hsa-miR-137, hsa-miR-143-3p, hsa-miR-200b-3p, hsa-miR-218-5p, and hsa-miR-3666), and three mRNAs (SMAD4, METTL3, and EGFR).

### Identification and validation of the diagnostic markers for OP

**Figure 8:**
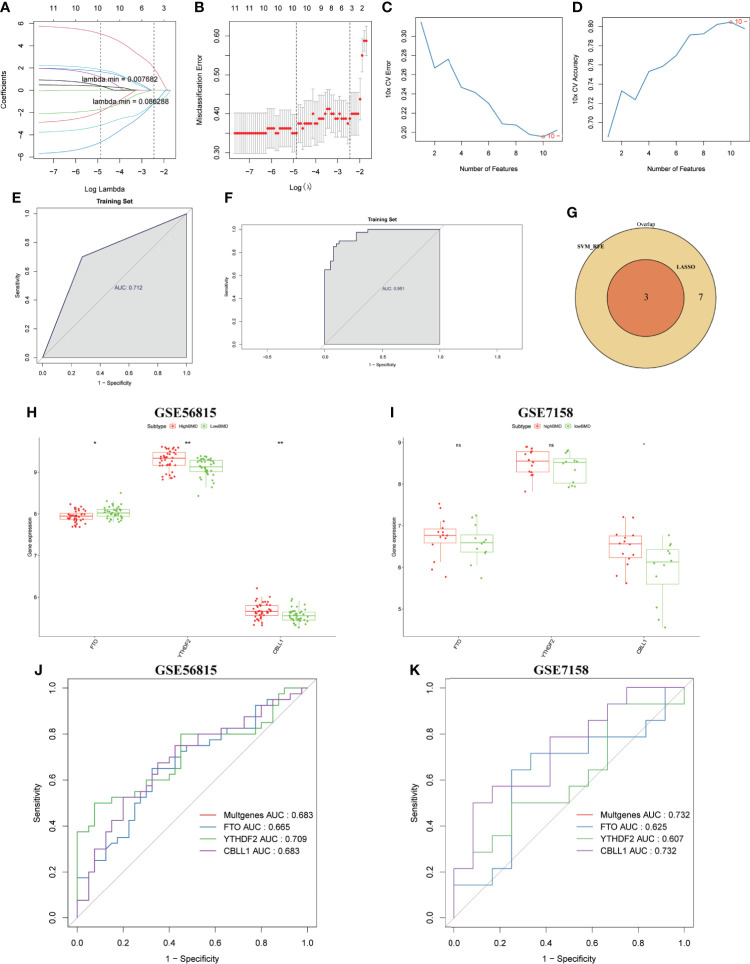
Identification and validation of the diagnostic markers for OP. **(A)** LASSO regression coefficient profiles of the 13 m6A regulators. Each curve represents the changing trajectory of each m6A regulator. **(B)** Tenfold cross-validation for optimal parameter selection in the LASSO model. Each red dot represents a lambda value with a confidence interval. The two dotted lines represent the values at minimum criteria and 1-standard error (1-SE) criteria by 10-fold cross-validation. The *x*-axis shows the penalization coefficient (log *λ*). The *y*-axis shows the partial likelihood deviance values with error bars. **(C)** The curve of the total within sum of squared error curve under corresponding cluster number k, and it reached the “elbow point” when k = 10. **(D)** The curve of average silhouette width under corresponding cluster number k, and the maximum of average silhouette width was achieved when k = 10. **(E, F)** ROC curves validated the performances of the LASSO regression model and the SVM-RFE model. **(G)** Venn plots show the candidate genes by overlapping the candidate genes selected from the LASSO regression model and the SVM-RFE model. **(H, I)** Boxplots showing the three differentially expressed m6A regulators (FTO, YTHDF2, and CBLL1) between high-BMD women and low-BMD women in GSE56815 and GSE7158 datasets. **(J, K)** ROC curves validated the performances of three m6A regulators (FTO, YTHDF2, and CBLL1) for the prediction of OP in GSE56815 and GSE7158 datasets.

Finally, we constructed a LASSO regression and SVM-RFE model to select the candidate m6A regulators from 13 DE-m6A regulators to predict the occurrence of OP. As shown in **Figures 8A** **, B**, an optimal *λ* was selected using 10-fold cross-validation based on the minimum mean square error. Three m6A regulators (FTO, YTHDF2, and CBLL1) were selected as the candidate genes by LASSO analysis. Moreover, the SVM-RFE model was used to narrow down 13 DE-m6A regulators, and 10 m6A regulators (YTHDF2, FTO, CBLL1, LRPPRC, YTHDF3, ZC3H13, RBM15B, FMR1, RBM15, and YTHDF1) were selected as the candidate genes (**Figures 8C** **, D**). Moreover, ROC curves were drawn to assess the predictive ability of LASSO regression and SVM-RFE models, and the AUC values of ROC for LASSO regression and SVM-RFE models were 0.712 and 0.951, respectively (**Figures 8E** **, F**), which indicated that LASSO regression and SVM-RFE models showed a high accuracy for OP prediction. Thus, three m6A regulators (FTO, YTHDF2, and CBLL1) were subsequently selected as the candidate genes for OP by overlapping the candidate genes from two models (**Figure 8G**). Finally, we also detected the predictive ability of three candidate genes in the training cohort (GSE56815) and validation cohort (GSE7158). As shown in **Figure 8H**, the expression of FTO was downregulated, whereas the expression of YTHDF2 and CBLL1 was upregulated in high-BMD women compared with low-BMD women in the GSE56815 dataset. CBLL1 has a higher expression in high-BMD women than in low-BMD women in the GSE7158 dataset (**Figure 8I**). Furthermore, the AUC values of ROC curves for three m6A regulators together (GSE56815, multigene, AUC = 0.683; GSE7158, multigene, AUC = 0.732) indicated the more powerful prediction for OP than the predictive ability of a unique gene of them (GSE56815, FTO, AUC = 0.665; YTHDF2, AUC = 0.709; CBLL1, AUC = 0.683; GSE7158, FTO, AUC = 0.625; YTHDF2, AUC = 0.607; CBLL1, AUC = 0.732) (**Figures 8J** **, K**).

## Discussion

OP is a degenerative bone disease that is characterized by depleted bone mass, destroyed bone structure, bone fragility, and fractures (39). Emerging evidence has indicated that m6A modification plays an important role in impaired bone information and maintained the balance of bone homeostasis of OP (40). However, the role and regulatory mechanism of m6A regulators in OP remains unclear. Here, we integrated analyses of the m6A-related molecular pattern, m6A targets and their related regulatory mechanism, and the m6A-related diagnostic model in OP. Firstly, we investigated the differential expression of 21 m6A regulators and m6A-related molecular pattern in OP, and 13 DE-m6A regulators, namely, METTL3, HNRNPC, FTO, LRPPRC, YTHDC1, YTHDF1, ZC3H13, RBM15, YTHDF3, FMR1, RBM15B, YTHDF2, and CBLL1, were found in OP. Based on these m6A regulators, 40 low-BMD women were distributed into two subclusters (cluster 1 = 23, and cluster 2 = 17). There were 5,260 significant m6A-related DEGs between two subclusters.

Then, we investigated the functional pathways of the m6A-related DEGs. These m6A-related DEGs were involved in OP-related signaling pathways, including apoptosis, cell cycle, immunity, lipid and glucose metabolism, methylation, and osteoclast differentiation. Moreover, 22 pathways were upregulated in cluster 2 compared to cluster 1, such as PI3K-Akt, MAPK, and immune-related pathways (regulation of monocyte differentiation, response to granulocyte-macrophage colony-stimulating factor, chemokine signaling pathways, positive regulation of T-cell apoptotic process, and B-cell receptor signaling pathway), bone formation and resorption (osteoclast proliferation and differentiation, and bone resorption), bone metabolism (glucocorticoid metabolic process, fructose and mannose metabolism, canonical glycolysis, lipid homeostasis, glycolysis/gluconeogenesis, pentose phosphate pathway, and carbon metabolism), and cell apoptosis (regulation of extrinsic apoptotic signaling pathway in the absence of ligand and engulfment of apoptosis cell). In contrast, the 22 pathways were upregulated in cluster 1 compared to cluster 2, mainly associated with cell cycle-related pathways (regulation of cell cycle, G2/M transition of mitotic cell cycle, mitotic cell cycle, mitotic cell cycle process, cell cycle checkpoint, regulation of mitotic metaphase/anaphase transition, negative regulation of cell cycle phase transition, negative regulation of cell cycle G2/M phase transition, and negative regulation of mitotic cell cycle phase transition). Other pathways included bone cell development, RNA or tRNA methylation, positive regulation of gluconeogenesis, fatty acid metabolism, tumor necrosis factor-mediated signaling pathway, T-cell receptor signaling pathway, regulation of telomere maintenance *via* telomerase, autophagy, apoptosis, and peroxisome.

A previous study has indicated that the PI3K-Akt signaling pathway has a protective function in glucocorticoid-induced OP (41), and activating the PI3K-Akt signaling pathway promotes cell adhesion, cell viability, and osteogenic differentiation (42). Nevertheless, inhibition of the MAPK signaling pathway can ameliorate OP (43, 44). Generally, osteoclasts are specialized cells derived from the monocyte and macrophage hematopoietic lineage that adhere to the bone matrix and degrade it (45). Altered cytokine expression and immune cell profile are often found in OP (14), which affect the communication between immune cells and osteoblasts and osteoclasts to regulate the processes of OP (46, 47). These findings suggested that the OP patients in cluster 2 might show the activated osteoclast and be involved in the immune-activated bone microenvironment. Furthermore, we also found several metabolism-related pathways involved in OP. Glucocorticoid is widely used to inhibit inflammation of the immune system (48), and it also is a risk factor for bone fragility that leads to OP (49, 50). Other metabolism-related pathways, including canonical glycolysis, glycolysis/gluconeogenesis, lipid homeostasis, pentose phosphate pathway, and carbon metabolism, were extremely rare in OP. Recent evidence indicates that active metabolic reprogramming involves osteoclastogenesis (51), which represents a therapeutic target for OP treatment (52). Here, we found that positive regulation of gluconeogenesis and fatty acid metabolism were upregulated in cluster 1. In addition, we also found a TNF-mediated signaling pathway and T-cell receptor signaling pathway involved in cluster 1. TNF-a plays an important role in immune responses and bone metabolism (15). Inhibition of TNF-a reduces osteoclast formation to suppress OP (53, 54). Moreover, several cell cycle-related pathways, autophagy, and apoptosis involved in cluster 1, osteoblast proliferation, cell cycle, apoptosis, and autophagy directly affect the OP process (55).

Thirdly, we further explored the m6A targets and their related regulatory mechanism in OP. We established a WGCNA to identify the OP-related hub modules and key genes. We identified five modules (MEblue, MEbrown, MEyellow, MEred, and MEturquoise) significantly associated with OP. In particular, the m6A targets were significantly enriched in module brown, then an m6A regulator–target gene–pathway network in module brown was constructed, which contained two m6A regulators (METTL3 and YTHDF2), four m6A targets (SMAD4, HIPK3, MAP4K3, and EGFR), and 19 signaling pathways, such as MAPK, Foxo, PI3K-Akt, focal adhesion, Rap1 signaling pathway, Ras, calcium, cellular senescence, and TGF-beta signaling pathways. METTL3 promotes the osteogenic potential of mesenchymal stem cells in OP (28, 56, 57), while YTHDF2 has rarely been found in OP. SAMD4 and EGFR play critical roles in osteoprogenitor maintenance and bone formation (58, 59). The above finding proved that the potential regulatory mechanism of m6A regulators modulated the process of OP. MiRNAs and lncRNAs are two targets that have recently come into the spotlight due to their regulatory ability to affect gene expression at the transcriptional or post-transcriptional levels and provide epigenetic modification (54, 60, 61). Here, we found that an m6A regulator (METTL3) and three m6A targets (SMAD4, HIPK3, and EGFR) might have a function in OP in an lncRNA–miRNA-dependent manner.

Finally, we constructed a diagnostic model and identified three m6A regulators (FTO, YTHDF2, and CBLL1) used to diagnose OP. We found FTO upregulated in OP, whereas YTHDF2 and CBLL1 were downregulated in OP. Previous studies have indicated that FTO inhibits osteogenic differentiation to promote OP (26, 62).

Although our study firstly discovered the function of m6A regulators, m6A-related molecular patterns, and diagnostic values of m6A regulators in OP, there are also some limitations here. The first limitation is the lack of complete clinical characteristics of OP patients in the original dataset. Moreover, although OP is a common disease for women, the sample size of OP remains small in the original dataset, and the expression of m6A regulators was verified in a small cohort. Therefore, future research needs a randomized control study with a large sample size to verify our results.

## Conclusion

Taken together, our findings indicated the function of m6A regulators in the OP process and diagnosis, which provided a novel insight into the pathologic analyses and diagnostic biomarker exploration at the cellular and molecular levels.

## Data availability statement

The original contributions presented in the study are included in the article/**Supplementary Material**. Further inquiries can be directed to the corresponding author/s.

## Author contributions

LD and JF conceived studies and provided supervision. QB, MS, and XS collected the data and performed the bioinformatics analysis. QB wrote the original manuscript, and QB and MS provided the edited version. XS, QL, HP, and ZQ completed the tables and figures, and interpreted the results from clinical perspectives. All authors contributed to the article and approved the submitted version.

## Funding

This study was supported by the Applied Basic Research Joint Project of Kunming Medical University 2019FE001 (-227).

## Conflict of interest

The authors declare that the research was conducted in the absence of any commercial or financial relationships that could be construed as a potential conflict of interest.

## Publisher’s note

All claims expressed in this article are solely those of the authors and do not necessarily represent those of their affiliated organizations, or those of the publisher, the editors and the reviewers. Any product that may be evaluated in this article, or claim that may be made by its manufacturer, is not guaranteed or endorsed by the publisher.

## References

[1] Salari N Darvishi N. Bartina Y Larti M Kiaei A Hemmati M. Global prevalence of osteoporosis among the world older adults: a comprehensive systematic review and meta-analysis. J Orthop Surg Res (2021) 16(1):669. doi: 10.1186/s13018-021-02821-8 34774085 PMC8590304

[2] Osteoporosis prevention, diagnosis, and therapy. Jama (2001) 285(6):785–95. doi: 10.1001/jama.285.6.785 11176917

[3] Kanis JA Melton LJ 3rd Christiansen C Johnston CC Khaltaev N. The diagnosis of osteoporosis. J Bone Miner Res (1994) 9(8):1137–41. doi: 10.1002/jbmr.5650090802 7976495

[4] Reid IR. A broader strategy for osteoporosis interventions. Nat Rev Endocrinol (2020) 16(6):333–9. doi: 10.1038/s41574-020-0339-7 32203407

[5] Ghaderi S Sahaf R Mohammadi Shahbalaghi F Ansari G Gharanjic A Ashrafi K. Prevalence of depression in elderly Kurdish community residing in boukan, Iran. (2012) 7(1):57–66.

[6] Cui L Jackson M. Wessler Z. Gitlin M. Xia W. Estimating the future clinical and economic benefits of improving osteoporosis diagnosis and treatment among women in China: a simulation projection model from 2020 to 2040. Arch Osteoporos (2021) 16(1):118. doi: 10.1007/s11657-021-00958-x 34338927

[7] Si L Winzenberg TM Chen M Jiang Q Palmer AJ. Residual lifetime and 10 year absolute risks of osteoporotic fractures in Chinese men and women. Curr Med Res Opin (2015) 31(6):1149–56. doi: 10.1185/03007995.2015.1037729 25851177

[8] Yu F Xia W. The epidemiology of osteoporosis, associated fragility fractures, and management gap in China. Arch Osteoporos (2019) 14(1):32. doi: 10.1007/s11657-018-0549-y 30848398

[9] Clynes MA Harvey NC Curtis EM Fuggle NR Dennison EM Cooper C. The epidemiology of osteoporosis. Br Med Bull (2020) 133(1):105–17. doi: 10.1093/bmb/ldaa005 PMC7115830 32282039

[10] Li N Zheng B Liu M Zhou H Zhao L Cai H. Cost-effectiveness of antiosteoporosis strategies for postmenopausal women with osteoporosis in China. Menopause (2019) 26(8):906–14. doi: 10.1097/GME.0000000000001339 30994577

[11] Rachner TD Khosla S Hofbauer LC. Osteoporosis: now and the future. Lancet (2011) 377(9773):1276–87. doi: 10.1016/S0140-6736(10)62349-5 PMC3555696 21450337

[12] Chen X Wang Z Duan N Zhu G Schwarz EM Xie C. Osteoblast-osteoclast interactions. Connect Tissue Res (2018) 59(2):99–107. doi: 10.1080/03008207.2017.1290085 28324674 PMC5612831

[13] Díaz López JB Rodríguez Rodríguez A Ramos B Caramelo C Rodríguez García M Cannata Andía JB. Osteoporosis, estrogens, and bone metabolism. implications for chronic renal insufficiency. Nefrologia (2003) 23 (Suppl 2):78–83. 12778860

[14] Fischer V Haffner-Luntzer M. Interaction between bone and immune cells: Implications for postmenopausal osteoporosis. Semin Cell Dev Biol (2022) 123:14–21. doi: 10.1016/j.semcdb.2021.05.014 34024716

[15] Wang T He C. TNF-α and IL-6: The link between immune and bone system. Curr Drug Targets (2020) 21(3):213–27. doi: 10.2174/1389450120666190821161259 31433756

[16] Wang X Guo B Li Q Peng J Yang Z Wang A. miR-214 targets ATF4 to inhibit bone formation. Nat Med (2013) 19(1):93–100. doi: 10.1038/nm.3026 23223004

[17] Chen H Hu B. Lv X Zhu S Zhen G Wan M. Prostaglandin E2 mediates sensory nerve regulation of bone homeostasis. Nat Commun (2019) 10(1):181. doi: 10.1038/s41467-018-08097-7 30643142 PMC6331599

[18] Yang K Miron RJ Bian Z Zhang YF. A bone-targeting drug-delivery system based on semaphorin 3A gene therapy ameliorates bone loss in osteoporotic ovariectomized mice. Bone (2018) 114:40–9. doi: 10.1016/j.bone.2018.06.003 29883786

[19] Wang X Lu Z Gomez A Hon GC Yue Y Han D. N6-methyladenosine-dependent regulation of messenger RNA stability. Nature (2014) 505(7481):117–20. doi: 10.1038/nature12730 PMC3877715 24284625

[20] Liu J Yue Y Han D Wang X Fu Y Zhang L. A METTL3-METTL14 complex mediates mammalian nuclear RNA N6-adenosine methylation. Nat Chem Biol (2014) 10(2):93–5. doi: 10.1038/nchembio.1432 PMC3911877 24316715

[21] Jia G Fu Y He C. Reversible RNA adenosine methylation in biological regulation. Trends Genet (2013) 29(2):108–15. doi: 10.1016/j.tig.2012.11.003 PMC3558665 23218460

[22] Liao S Sun H Xu C. YTH domain: A family of N(6)-methyladenosine (m(6)A) readers. Genomics Proteomics Bioinf (2018) 16(2):99–107. doi: 10.1016/j.gpb.2018.04.002 PMC6112328 29715522

[23] Zhao BS Roundtree IA He C. Post-transcriptional gene regulation by mRNA modifications. Nat Rev Mol Cell Biol (2017) 18(1):31–42. doi: 10.1038/nrm.2016.132 27808276 PMC5167638

[24] Alarcón CR Lee H Goodarzi H Halberg N Tavazoie SF. N6-methyladenosine marks primary microRNAs for processing. Nature (2015) 519(7544):482–5. doi: 10.1038/nature14281 PMC4475635 25799998

[25] Sun Z Wang H Wang Y Yuan G Yu X Jiang H. MiR-103-3p targets the m(6) a methyltransferase METTL14 to inhibit osteoblastic bone formation. Aging Cell (2021) 20(2):e13298. doi: 10.1111/acel.13298 33440070 PMC7884043

[26] Wang J Fu Q Yang J Liu JL Hou SM Huang X. RNA N6-methyladenosine demethylase FTO promotes osteoporosis through demethylating Runx2 mRNA and inhibiting osteogenic differentiation. Aging (Albany NY) (2021) 13(17):21134–41. doi: 10.18632/aging.203377 PMC8457567 34496349

[27] Chen LS Zhang M Chen P Xiong XF Liu PQ Wang HB. The m(6)A demethylase FTO promotes the osteogenesis of mesenchymal stem cells by downregulating PPARG. Acta Pharmacol Sin (2022) 43(5):1311–23. doi: 10.1038/s41401-021-00756-8 PMC9061799 34462564

[28] Peng J Zhan Y Zong Y. METTL3-mediated LINC00657 promotes osteogenic differentiation of mesenchymal stem cells via miR-144-3p/BMPR1B axis. Cell Tissue Res (2022) 388(2):301–12. doi: 10.1007/s00441-022-03588-y 35192037

[29] Monti S Tamayo P Mesirov J Golub TJMl. Consensus clustering: a resampling-based method for class discovery and visualization of gene expression microarray data. (2003) 52(1):91–118.

[30] Li W Cerise JE Yang Y Han H. Application of t-SNE to human genetic data. J Bioinform Comput Biol (2017) 15(4):1750017. doi: 10.1142/S0219720017500172 28718343

[31] Subramanian A Tamayo P Mootha VK Mukherjee S Ebert BL Gillette MA. Gene set enrichment analysis: a knowledge-based approach for interpreting genome-wide expression profiles. Proc Natl Acad Sci U.S.A. (2005) 102(43):15545–50. doi: 10.1073/pnas.0506580102 PMC1239896 16199517

[32] Langfelder P Horvath S. WGCNA: an r package for weighted correlation network analysis. BMC Bioinf (2008) 9:559. doi: 10.1186/1471-2105-9-559 PMC2631488 19114008

[33] Deng S Zhang H Zhu K Li X Ye Y Li R. M6A2Target: a comprehensive database for targets of m6A writers, erasers and readers. Brief Bioinform (2021) 22(3):bbaa055. doi: 10.1093/bib/bbaa055 32392583

[34] Cao J Zhang S. A Bayesian extension of the hypergeometric test for functional enrichment analysis. Biometrics (2014) 70(1):84–94. doi: 10.1111/biom.12122 24320951 PMC3954234

[35] Jeggari A Marks DS Larsson E. miRcode: a map of putative microRNA target sites in the long non-coding transcriptome. Bioinformatics (2012) 28(15):2062–3. doi: 10.1093/bioinformatics/bts344 PMC3400968 22718787

[36] Tibshirani R. Regression shrinkage and selection via the lasso. J R Stat Soc (1996) 58(1):267–88. doi: 10.1111/j.2517-6161.1996.tb02080.x

[37] Sanz H Valim C Vegas E Oller JM Reverter F. SVM-RFE: selection and visualization of the most relevant features through non-linear kernels. BMC Bioinf (2018) 19(1):432. doi: 10.1186/s12859-018-2451-4 PMC6245920 30453885

[38] Huang ML Hung YH Lee WM Li RK Jiang BR. SVM-RFE based feature selection and taguchi parameters optimization for multiclass SVM classifier. ScientificWorldJournal (2014) 2014:795624. doi: 10.1155/2014/795624 25295306 PMC4175386

[39] Wicklein S Gosch M. [Osteoporosis and multimorbidity]. Z Gerontol Geriatr (2019) 52(5):433–9. doi: 10.1007/s00391-019-01569-5 31214779

[40] Zou Z He T Liu Y Zheng L Zhong Y Mo Y. Emerging role of m6A modification in osteogenesis of stem cells. J Bone Miner Metab (2022) 40(2):177–88. doi: 10.1007/s00774-021-01297-0 35091784

[41] Ge X Zhou G. Protective effects of naringin on glucocorticoid-induced osteoporosis through regulating the PI3K/Akt/mTOR signaling pathway. Am J Transl Res (2021) 13(6):6330–41. PMC8290725 34306372

[42] Wang J Ma XY Feng YF Ma ZS Ma TC Zhang Y. Magnesium ions promote the biological behaviour of rat calvarial osteoblasts by activating the PI3K/Akt signalling pathway. Biol Trace Elem Res (2017) 179(2):284–93. doi: 10.1007/s12011-017-0948-8 28205079

[43] Zheng S Wang YB Yang YL Chen BP Wang CX Li RH. LncRNA MALAT1 inhibits osteogenic differentiation of mesenchymal stem cells in osteoporosis rats through MAPK signaling pathway. Eur Rev Med Pharmacol Sci (2019) 23(11):4609–17. doi: 10.26355/eurrev_201906_18038. 31210287

[44] Lin Z. Polydatin ameliorates osteoporosis via suppression of the mitogen-activated protein kinase signaling pathway. Front Cell Dev Biol (2021) 9:730362. doi: 10.3389/fcell.2021.730362 34660587 PMC8511501

[45] Boyle WJ Simonet WS Lacey DL. Osteoclast differentiation and activation. Nature (2003) 423(6937):337–42. doi: 10.1038/nature01658 12748652

[46] Saxena Y Routh S Mukhopadhaya A. Immunoporosis: Role of innate immune cells in osteoporosis. Front Immunol (2021) 12:687037. doi: 10.3389/fimmu.2021.687037 34421899 PMC8374941

[47] Brylka LJ Schinke T. Chemokines in physiological and pathological bone remodeling. Front Immunol (2019) 10:2182. doi: 10.3389/fimmu.2019.02182 31572390 PMC6753917

[48] Chotiyarnwong P McCloskey EV. Pathogenesis of glucocorticoid-induced osteoporosis and options for treatment. Nat Rev Endocrinol (2020) 16(8):437–47. doi: 10.1038/s41574-020-0341-0 32286516

[49] Ward LM. Glucocorticoid-induced osteoporosis: Why kids are different. Front Endocrinol (Lausanne) (2020) 11:576. doi: 10.3389/fendo.2020.00576 33391179 PMC7772619

[50] Frenkel B White W Tuckermann J. Glucocorticoid-induced osteoporosis. Adv Exp Med Biol (2015) 872:179–215. doi: 10.1007/978-1-4939-2895-8_8 26215995 PMC5905346

[51] Park-Min KH. Metabolic reprogramming in osteoclasts. Semin Immunopathol (2019) 41(5):565–72. doi: 10.1007/s00281-019-00757-0 PMC7671717 31552471

[52] Taubmann J Krishnacoumar B Böhm C Faas M Müller DIH Adam S. Metabolic reprogramming of osteoclasts represents a therapeutic target during the treatment of osteoporosis. Sci Rep (2020) 10(1):21020. doi: 10.1038/s41598-020-77892-4 33273570 PMC7713370

[53] Zha L He L Liang Y Qin H Yu B Chang L. TNF-α contributes to postmenopausal osteoporosis by synergistically promoting RANKL-induced osteoclast formation. BioMed Pharmacother (2018) 102:369–74. doi: 10.1016/j.biopha.2018.03.080 29571022

[54] Yu H Zhou W Yan W Xu Z Xie Y Zhang P. LncRNA CASC11 is upregulated in postmenopausal osteoporosis and is correlated with TNF-α. Clin Interv Aging (2019) 14:1663–9. doi: 10.2147/CIA.S205796 PMC6759792 31571846

[55] Tang N Zhao H Zhang H Dong Y. Effect of autophagy gene DRAM on proliferation, cell cycle, apoptosis, and autophagy of osteoblast in osteoporosis rats. J Cell Physiol (2019) 234(4):5023–32. doi: 10.1002/jcp.27304 30203495

[56] Wu Y Xie L Wang M Xiong Q Guo Y Liang Y. Mettl3-mediated m(6)A RNA methylation regulates the fate of bone marrow mesenchymal stem cells and osteoporosis. Nat Commun (2018) 9(1):4772. doi: 10.1038/s41467-018-06898-4 30429466 PMC6235890

[57] Wu T. METTL3-m(6) a methylase regulates the osteogenic potential of bone marrow mesenchymal stem cells in osteoporotic rats via the wnt signalling pathway. Cell Prolif (2022) 55(5):e13234. doi: 10.1111/cpr.13234 35470497 PMC9136513

[58] Liang W Lin M Li X Li C Gao B Gan H. Icariin promotes bone formation via the BMP-2/Smad4 signal transduction pathway in the hFOB 1.19 human osteoblastic cell line. Int J Mol Med (2012) 30(4):889–95.doi: 10.3892/ijmm.2012.1079 22842877

[59] Liu G Xie Y Su J Qin H Wu H Li K. The role of EGFR signaling in age-related osteoporosis in mouse cortical bone. FASEB J (2019) 33(10):11137–47. doi: 10.1096/fj.201900436RR 31298955

[60] Yang X Yang J Lei P Wen T. LncRNA MALAT1 shuttled by bone marrow-derived mesenchymal stem cells-secreted exosomes alleviates osteoporosis through mediating microRNA-34c/SATB2 axis. Aging (Albany NY) (2019) 11(20):8777–91. doi: 10.18632/aging.102264 PMC6834402 31659145

[61] Han J Kong H Wang X Zhang XA. Novel insights into the interaction between N6-methyladenosine methylation and noncoding RNAs in musculoskeletal disorders. Cell Prolif (2022):e13294. doi: 10.1111/cpr.13294 35735243 PMC9528765

[62] Zhang X Wang Y Zhao H Han X Zhao T Qu P. Extracellular vesicle-encapsulated miR-22-3p from bone marrow mesenchymal stem cell promotes osteogenic differentiation via FTO inhibition. Stem Cell Res Ther (2020) 11(1):227. doi: 10.1186/s13287-020-01707-6 32522250 PMC7285613

