# Supplementary figures and images for: Comprehensive analysis of the m6A-related molecular patterns and diagnostic biomarkers in osteoporosis
Source: Front Endocrinol (Lausanne). 2022 Aug 10;13:957742. doi: 10.3389/fendo.2022.957742 (PMC9399504; doi:10.3389/fendo.2022.957742)

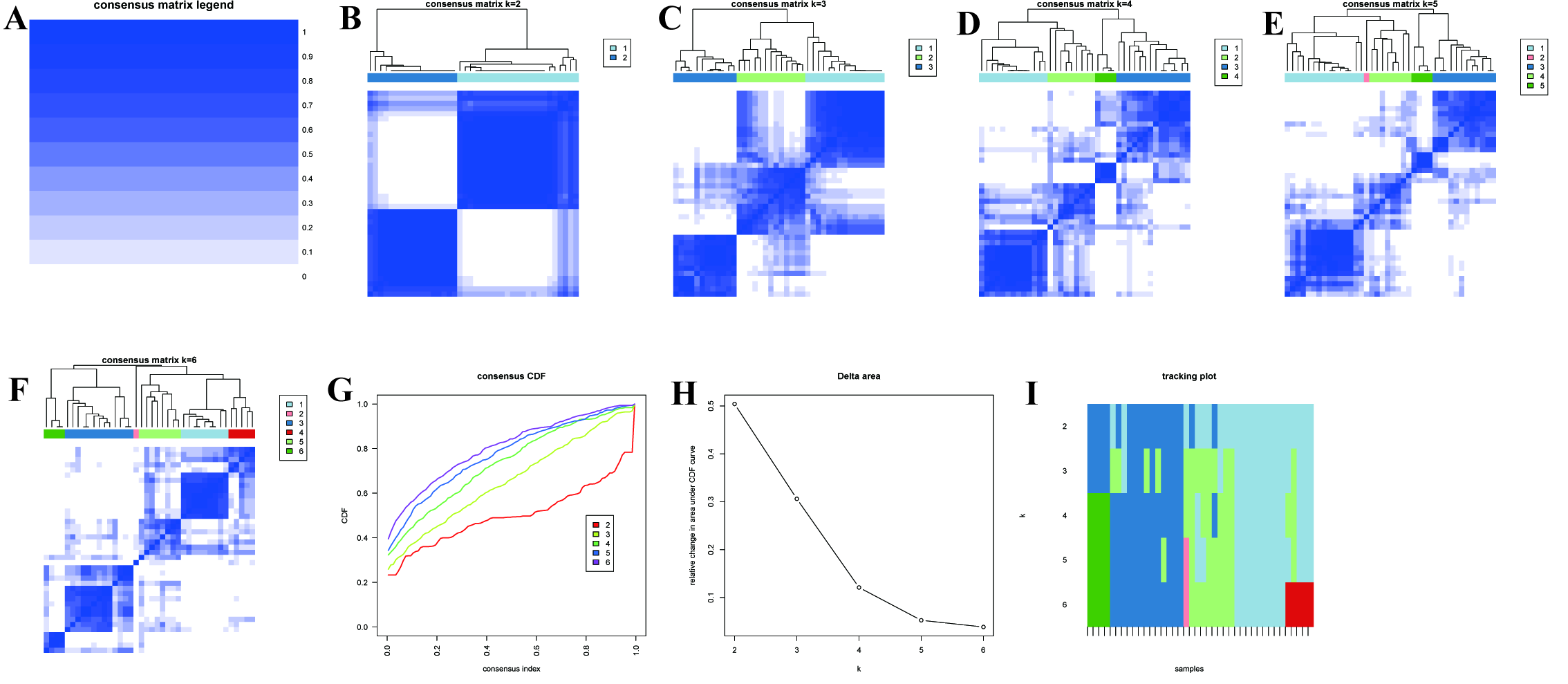

Supplement: Supplementary file 1 [file Image_1.tif]

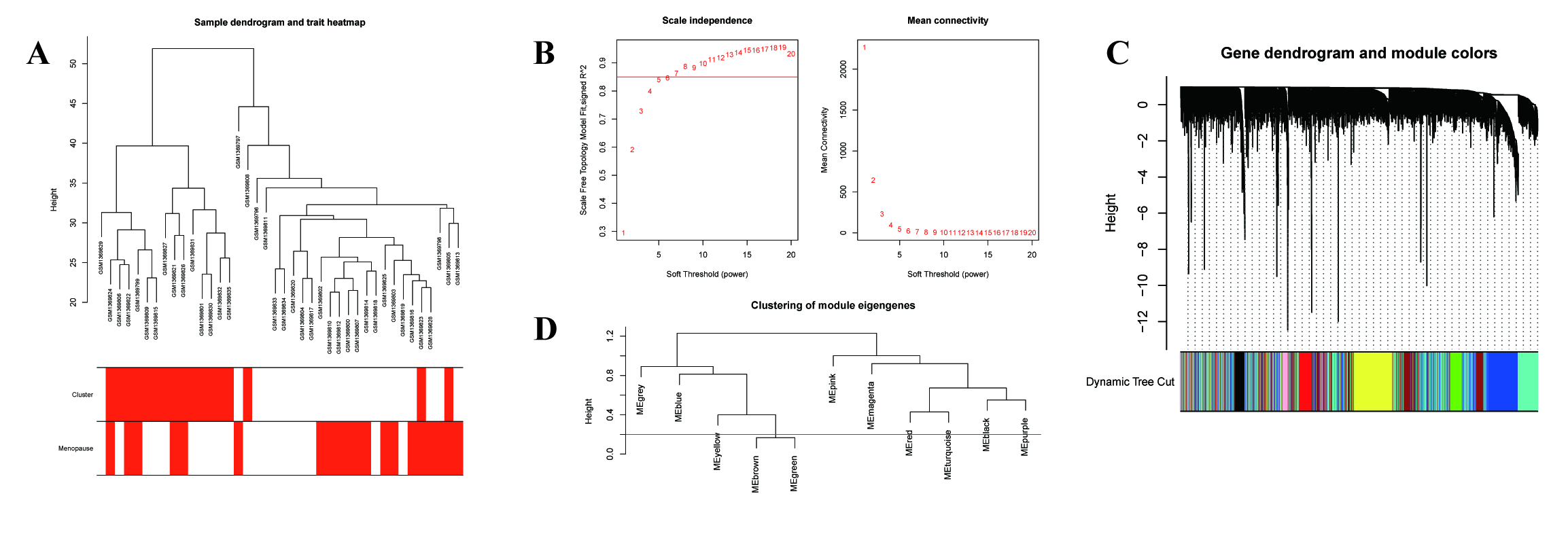

Supplement: Supplementary file 2 [file Image_2.tif]
