# Supplementary material for: Comprehensive analysis of the m6A-related molecular patterns and diagnostic biomarkers in osteoporosis
Source: Front Endocrinol (Lausanne). 2022 Aug 10;13:957742. doi: 10.3389/fendo.2022.957742 (PMC9399504; doi:10.3389/fendo.2022.957742)
Supplement: Supplementary file 4 [file Presentation_1.pdf]

Figure S1 Consensus clustering analysis of the 40 low-BMD women based on the expression of 13 DE-m6A regulators.

(A)-(F) The consensus clustering matrix for k = 2-6.

(G) The cumulative distribution function (CDF) of consensus clustering for k from 2 to 6.

(H) Relative change in area under the CDF curve for k from 2 to 6.

(I) The tracking plot presented to verify the principal component for k from 2 to 6.

Figure S2 Construction of the WGCNA.

(A) Clustering dendrogram of samples with trait heatmap.

(B) Detection of the scale-free index for various soft-threshold powers (β). Left panel, X-axis represented a function of soft-threshold power, and Y-axis represented the scale-free fit index. Right panel, X-axis represented a function of soft-threshold power, and Y-axis represented the mean connectivity (degree).

(C) Eigengene dendrogram of samples.

(D) Dendrogram of the genes based on the measurement of dissimilarity (1-TOM).
